# Linkage disequilibrium maps for European and African populations constructed from whole genome sequence data

**DOI:** 10.1038/s41597-019-0227-y

**Published:** 2019-10-17

**Authors:** Alejandra Vergara-Lope, M. Reza Jabalameli, Clare Horscroft, Sarah Ennis, Andrew Collins, Reuben J. Pengelly

**Affiliations:** Human Genetics & Genomic Medicine, Faculty of Medicine, University of Southampton, Southampton, UK

**Keywords:** Genomics, Anthropology, Population genetics

## Abstract

Quantification of linkage disequilibrium (LD) patterns in the human genome is essential for genome-wide association studies, selection signature mapping and studies of recombination. Whole genome sequence (WGS) data provides optimal source data for this quantification as it is free from biases introduced by the design of array genotyping platforms. The Malécot-Morton model of LD allows the creation of a cumulative map for each choromosome, analogous to an LD form of a linkage map. Here we report LD maps generated from WGS data for a large population of European ancestry, as well as populations of Baganda, Ethiopian and Zulu ancestry. We achieve high average genetic marker densities of 2.3–4.6/kb. These maps show good agreement with prior, low resolution maps and are consistent between populations. Files are provided in BED format to allow researchers to readily utilise this resource.

## Abstract (summary)

**Table:** 

Measurement(s)	Linkage Disequilibrium
Technology Type(s)	whole genome sequencing
Factor Type(s)	ethnic group
Sample Characteristic - Organism	Homo sapiens
Sample Characteristic - Location	Europe • Africa

Machine-accessible metadata file describing the reported data: 10.6084/m9.figshare.9901838

## Background & Summary

Mapping of linkage disequilibrium (LD) is invaluable for many endeavours including identifying signatures of selection, refinement of signals in genome-wide association studies and studies into recombination^1–3^.

One approach to the quantification of LD is the generation of LD maps applying the Malécot-Morton model^4,5^. The product generated utilising the Malécot-Morton model are maps in cumulative linkage disequilibrium units (LDU), which are broadly analogous to an LD-based form of centimorgans. Previous studies have reported maps generated from array based genotyping data in multiple populations (e.g.^6^), allowing for cross-population comparisons.

The mathematical basis of *LDMAP* has been previously described^4,5^. In brief, *LDMAP* generates a cumulative map of LD distances between markers, based upon the Malécot-Morton model of association by distance:

1 $$\widehat{\rho }=(1-L)M{e}^{-\epsilon d}+L$$

where $\widehat{\rho }$ is the association between two markers in a population, *L* is the component of $\widehat{\rho }$ not due to LD, but due to confounding factors such as recent founder effects, *M* is the association at 0 distance (approximately 1 for monophyletic haplotypes), $\epsilon$ is the rate of decline in the association between the markers and *d* is the physical distance between the markers^5^. The final LDU map is built by cumulative addition of $\epsilon d$ for each inter-marker span.

The increasing availability of whole genome sequencing (WGS) data allows the investigation of LD patterns at the highest level, without the impact of issues such as ascertainment bias in the selection of single nucleotide polymorphism (SNP) markers. We have previously shown that WGS-based maps provide tangible benefits in their practical application. Arrays have been designed to give a reasonable coverage of LD information for a reduced set of SNPs, as such they have limited resolution and population-specific biases are introduced during SNP selection. Given that WGS variant identification is ‘hypothesis free’ (i.e. SNPs are not required to be pre-defined as in array genotyping), these data, and thus these maps, represent a maximally informative resource^7^.

The lack of ascertainment bias for SNP data collection is particularly important for African populations, as they have the greatest population diversity and are often under-represented in genomic studies. Though they are often underrepresented, these populations are particularly informative for many studies, given the extended time since a population bottleneck^7,8^. Higher resolution maps allow for analyses on a finer scale of the patterns of LD, such as structure within genes^9^.

Here, we report our generation of WGS based LD maps for four populations, one of European and three of African descent. These maps provide a valuable population genetic resource, providing a maximal resolution, selection bias free, dataset for studies which require the incorporation of LD statistics.

## Methods

Autosomal WGS data from two cohort sequencing studies was utilised. African populations were sequenced within the African Genome Diversity Project^8,10^, utilising Illumina short read sequencing to an average depth of 4x. European ancestry individuals were sequenced by the Wellderly Study^11^, utilising Complete Genomics high depth sequencing. Multidimensional scaling as implemented in *PLINK*^12^ was applied to ensure genetic homogeneity within the sub-cohorts.

SNPs were subject to quality control prior to map generation. Specifically, they were required to have a minor allele frequency ≥1%, <5% genotype missingness and not to significantly deviate from Hardy-Weinberg equilibrium (at α = 10^−3^). All analyses were undertaken using the reference genome GRCh37 (hg19).

LD maps were made using *LDMAP* with default parameters. Owing to the computational intensity of LD map generation, this was performed for 12,000 marker overlapping segments, which were then concatenated into full chromosome maps, removing the 25 terminal markers of each segment to avoid end effects.

## Data Records

LD maps reported here are freely available at 10.6084/m9.figshare.7850882 ^13^. These data are in Browser Extensible Data (BED) format, including the cumulative LDU position of every SNP marker within the generated maps. Additionally, these data are also made available as the kb/LDU ratio for each inter-SNP span providing a view of the regional ‘intensity’ of LD.

For the African populations^8^, 95–100 individuals were utilised for each sub-population, yielding approximately 14 million SNP markers (Table 1). The European map utilised 454 individuals^11^, yielding approximately 7.5 million markers. The increased population diversity for the African compared to European population can be seen in the increase common SNP density, as well as the longer LDU length which corresponds to the longer total haplotypic diversity within a population.

**Table 1:** Key statistics for generated LD maps.

Population	Individuals	Marker count	Density^a^	LDU
Baganda	100	13,439,201	4.35	129,640
Ethiopian	95	13,892,209	4.48	107,001
European	454	7,062,420	2.28	63,427
Zulu	100	14,205,839	4.59	130,156

^a^Average SNP markers per kb.

## Technical Validation

**Fig. 1:**
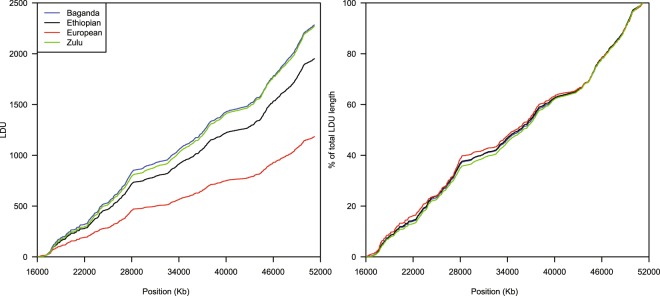
Comparison of the four maps for chromosome 22. The raw cumulative maps are shown (left), as well as maps normalised to have the same total length (right). It can be seen that the contour profiles of the maps are highly similar, though there is variation in the total map length.

**Fig. 2:**
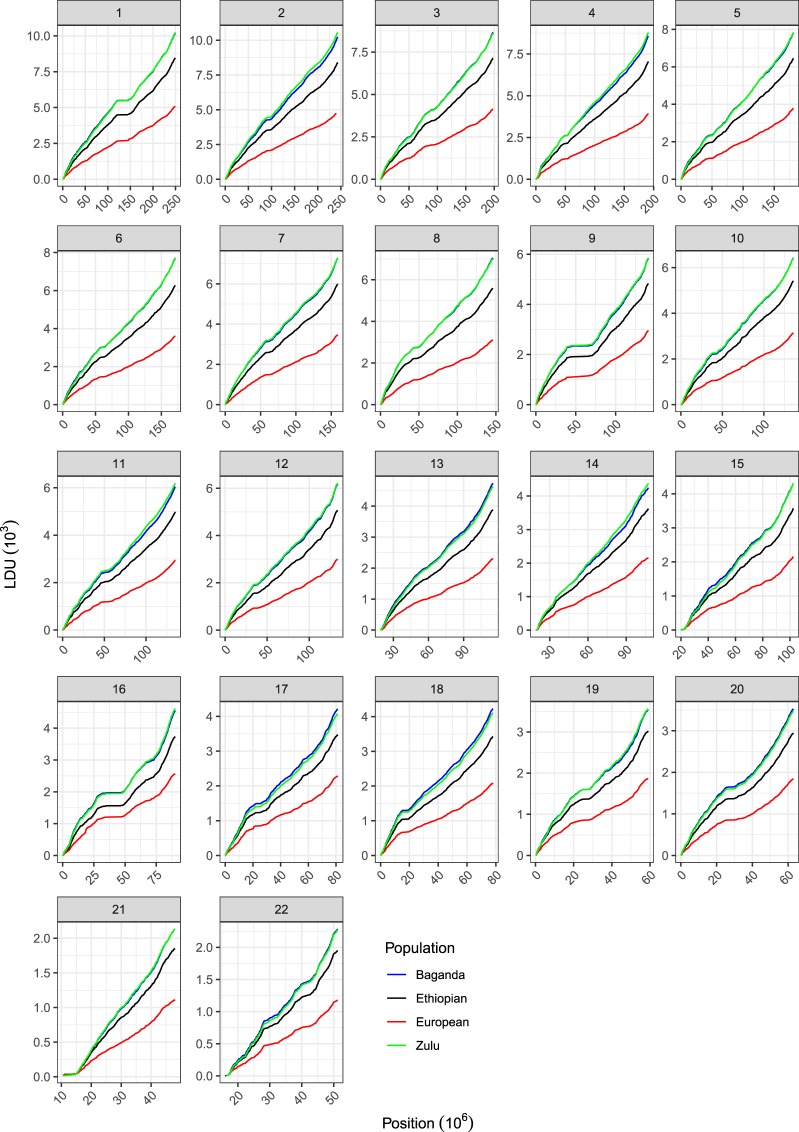
Comparison of the four maps for all autosomes. The raw cumulative maps are shown. It can be seen that the contour profiles of the maps are highly similar, with a consistend trend in LDU lengths for the populations, with European being consistently the shortest and Baganda/Zulu the longest.

For these data, we can determine that they are robust as they are consistent with prior, lower resolution maps, and that they are consistent between populations assessed (Figs 1 and 2). As we know that patterns of recombination and thus LD are broadly consistent between populations, this meets our prior expectations; furthermore the total map lengths are proportional to time since an effective population bottleneck (being longer in African populations reflecting the additional diversity present)^6,7,14^.

## Usage Notes

Maps can be readily incorporated into genomic analyses using tools such as BEDTools^15^, allowing annotation of regions with LD information for subsequent analysis such as determining whether a genomic feature has higher LD than background on average.

Genome wide association studies using a composite likelihood model can be undertaken with LD information as provided here, allowing for additional power for signal detection and refinement^2,16^.

## Data Availability

The core *LDMAP* software is written in C, and made available at www.soton.ac.uk/genomicinformatics/research/ld.page.
